# FlbD: A Regulator of Hyphal Growth, Stress Resistance, Pathogenicity, and Chlamydospore Production in the Nematode-Trapping Fungus *Arthrobotrys flagrans*

**DOI:** 10.3390/microorganisms13081847

**Published:** 2025-08-07

**Authors:** Yu Zhang, Shun-Qiao Peng, Wang-Ting He, Fei-Fei Gao, Qian-Fei Shi, Guo-Hong Li

**Affiliations:** State Key Laboratory for Conservation and Utilization of Bio-Resources in Yunnan, and Key Laboratory for Microbial Resources of the Ministry of Education, School of Life Sciences, Yunnan University, Kunming 650091, China; yuzhangqbl@163.com (Y.Z.); pengshunqiao@163.com (S.-Q.P.); hwt348432061@163.com (W.-T.H.); 17834459104@163.com (F.-F.G.); shiqf1201@163.com (Q.-F.S.)

**Keywords:** *Arthrobotrys flagrans*, AfFlbD, pathogenicity, chlamydospores

## Abstract

*Arthrobotrys flagrans* is a typical nematode-trapping fungus that captures nematodes by producing three-dimensional networks. FlbD is a DNA-binding protein containing a Myb domain, which plays a significant role in fungal development. However, the biological function of FlbD in nematode-trapping fungi remains unknown. In this study, we analyzed the physicochemical properties and conserved domains of AfFlbD and constructed the *AfFlbD* knockout strains (Δ*AfFlbD*) using homologous recombination. Our functional analysis revealed that the mutants produced more cottony aerial mycelia at the colony center. Additionally, the cell length of the mutants was reduced, indicating that AfFlbD regulates cell morphology in *A. flagrans*. Chemical stress tolerance assays of the mutants demonstrated reduced sensitivity to NaCl and sorbitol stresses but increased sensitivity to SDS and H_2_O_2_ stresses compared to the WT strain. Interestingly, the mutants spontaneously produced traps, and its pathogenicity to nematodes was significantly enhanced, suggesting that AfFlbD negatively regulates the pathogenicity of *A. flagrans*. Furthermore, the number of chlamydospores produced by the mutants was markedly reduced, though their morphology remained unchanged. Fluorescence localization analysis showed that AfFlbD localizes to the nuclei of chlamydospores, thereby regulating chlamydospore formation. This study provides important theoretical insights into the biological function of the FlbD transcription factor and offers new perspectives for the application of nematode-trapping fungi as a method of controlling plant-parasitic nematodes.

## 1. Introduction

Spores, whether sexual or asexual, constitute a crucial mode of reproduction for filamentous fungi. Furthermore, certain fungi rely on their pathogenicity to acquire nutrients from hosts during growth and development, thereby facilitating population propagation [1]. Both spore formation and the pathogenic process are precisely regulated by multiple genes. FlbA-E are upstream developmental activators of the central regulatory pathway for asexual sporulation in various fungi, governing the formation of asexual spores [2,3]. Of these activators, the *flbD* gene encodes a Myb-type DNA-binding protein, a critical transcription factor required to activate the expression of the *brlA* gene. FlbD has been reported in fungi such as *Saccharomyces cerevisiae*, *Aspergillus nidulans*, *A. fumigatus*, and *Magnaporthe oryzae*, where it has been found to profoundly influence the formation of conidia [4,5]. In *A. nidulans*, FlbD activates transcription of the *brlA* gene, thereby promoting conidiation. Deletion of this gene delays asexual sporulation and results in a “fluffy” colony phenotype [6]. In *Fusarium graminearum*, FlbD ensures proper hyphal differentiation and growth by regulating the expression of relevant genes. The deletion of *FlbD* reduces conidia production and impairs fungal pathogenicity [7]. Collectively, FlbD serves critical functions in regulating development and life cycle progression across diverse fungi. However, its biological role in nematode-trapping fungi remains uncharacterized.

*Arthrobotrys flagrans* is a representative nematode-trapping (NT) fungus that captures nematodes via three-dimensional adhesive networks and produces chlamydospores, making it one of the most promising biocontrol agents among nematophagous fungi [8,9]. Key components contributing to its trap formation and nematode capture include the *sofT* gene [10], the *sipC* gene [11], the secondary metabolite 6-methylsalicylic acid (6-MSA) [12], the small secreted protein CyrA [13], and the virulence factor NipA [14]. Deletion of the global regulator *AfLaeA* (a histone methyltransferase) impairs fungal growth, secondary metabolism, and pathogenicity toward nematodes, with the mutant notably losing the ability to produce chlamydospores [9]. The G protein-coupled receptor GprC, localized to the plasma membrane and mitochondria, perceives nematode pheromones (ascarosides) and activates mitochondrial function during nematode predation [8]. Biocontrol agents based on *A. flagrans* chlamydospores (e.g., Bioverm^®^) have been successfully applied to control nematodes in various livestock species [15]. Furthermore, *A. flagrans* exhibits significant biocontrol potential against *Meloidogyne* spp. [16] and *Xiphinema index* [10,17], establishing it as a highly promising biocontrol fungus for sustainable agriculture [18]. The major difference between *A. flagrans* and other nematode-trapping fungi is the ability of *A. flagrans* to readily form chlamydospores for survival [10]. However, the genes regulating chlamydospore formation in *A. flagrans* remain poorly understood.

To explore whether AfFlbD is involved in the chlamydospore formation and pathogenicity, we knocked out the *AfFlbD* gene in *A. flagrans* and conducted comparative analyses between the *AfFlbD* gene knockout strains (Δ*AfFlbD*) and the wild-type (WT) strain. Key differences were assessed in hyphal growth, stress responses, pathogenicity, and chlamydospore formation. Subcellular localization of AfFlbD was determined using GFP fluorescence. These investigations reveal the regulatory roles of AfFlbD in hyphal growth, stress response, pathogenicity, and chlamydospore formation in *A. flagrans*. Notably, AfFlbD functions as a negative regulator of trap development while serving as a positive regulator of chlamydospore formation. This study provides novel insights into the dual functionality of FlbD in chlamydospore formation and pathogenicity, revealing its contributions to both chlamydospore development and predatory mechanisms in nematode-trapping fungi.

## 2. Materials and Methods

### 2.1. Strains and Culture Conditions

*A. flagrans* and *Caenorhabditis elegans* were stored in the Microbial Library of the Germplasm Bank of Wild Species from Southwest China. The WT strain of *A. flagrans* was cultivated on potato dextrose agar (PDA) medium at 28 °C [9]. The *AfFlbD* gene knockout strains were cultivated on PDA medium containing 100 mg/mL hygromycin B.

### 2.2. Gene Structure and Conserved Protein Domain Analysis of AfFlbD

We extracted the *AfFlbD* gene sequence from the *A. flagrans* genome data [9]. The gene structure of AfFlbD was analyzed using the online tool Gene Structure Display Server (http://gsds.cbi.pku.edu.cn/, accessed on 5 June 2025) [9,19]. The molecular weight and isoelectric point (pI) of the AfFlbD protein were predicted with the Expasy ProtParam tool (https://web.expasy.org/protparam/, accessed on 1 June 2025). Conserved protein domains were identified using the NCBI CD-Search tool (https://www.ncbi.nlm.nih.gov/Structure/cdd/wrpsb.cgi, accessed on 1 June 2025). Phylogenetic tree construction and analysis were performed using MEGA X software (version 64-bit) [20]. Subcellular localization was predicted with Uniprot (https://www.uniprot.org/, accessed on 1 June 2025) [21].

### 2.3. Genomic DNA Extraction from A. flagrans

The WT strain of *A. flagrans* was inoculated on PDA medium and incubated at 28 °C for 4–5 days. Mycelia were harvested using sterile pipette tips, flash-frozen in liquid nitrogen, and thoroughly ground in a mortar. Genomic DNA was extracted using a Fungal DNA Kit (Magen, Guangzhou, China) [9].

### 2.4. Construction of the Gene Knockout Vector

Based on the *A. flagrans* genome data [9], upstream and downstream homologous arms flanking the *AfFlbD* gene were selected. Primers were designed using CE Design Software (https://tool.vazyme.com:18002/cetool/simple.html, accessed on 6 June 2024). The homologous arms were amplified using genomic DNA as the template, while the hygromycin resistance gene (*hph*) was amplified from the pCSN44 plasmid [22]. The fragments (upstream arm, *hph*, downstream arm) were directionally assembled into the pUC19 vector using a ClonExpress Ultra One Step Cloning Kit (Vazyme, Nanjing, China).

### 2.5. Protoplast Preparation of A. flagrans

The WT strain was inoculated on PDA medium and incubated at 28 °C for 3–4 days. Mycelial plugs were transferred to PDB medium and cultured under conditions of 28 °C, and 180 rpm for 24 h. Hyphae were filtered through sterile filter paper and rinsed with STC buffer (1 M sorbitol, 50 mM CaCl_2_, 10 mM Tris-HCl). Harvested hyphae were digested in 15–20 mL of filter-sterilized cell wall lysis enzyme solution under conditions of 28 °C, and 90 rpm for 4–5 h. Protoplasts were filtered through sterile filter paper and collected using centrifugation (4 °C, 3000× *g*, 8 min). Subsequently, 6–10 μg DNA was added to 10^7^ protoplasts. After 30 min incubation on ice, 1 mL PTC buffer (10 mM Tris-HCl [pH 7.5], 50 mM CaCl_2_, 50% [wt/vol] polyethylene glycol [PEG] 3350) was added, mixed gently by pipetting, and incubated at 28 °C for 40 min. The mixture was then plated onto PDASS regeneration medium (PDA supplement with 0.6 M of sucrose, 0.3 g/L peptone, 0.3 g/L tryptone, 0.3 g/L yeast extract, 8 g/L agar) in 9 cm dishes. Plates were sealed and incubated at 28 °C for 48 h, and transformants were selected on PDA supplemented with hygromycin B (100 μg/mL) or G418 (50 μg/mL) [9].

### 2.6. Screening and Verification of AfFlbD Knockout Strains

Transformants were cultured on PDA medium containing hygromycin B (100 μg/mL). Hyphae (~1.0 g) were scraped using sterile tips, mixed with quartz sand and urea extraction buffer, and homogenized in a high-throughput tissue grinder. Genomic DNA was extracted using a DNA isolation kit (Magen, Guangzhou, China). Gene-specific primers (F1/R1) amplified the target gene, while F2/R3 amplified the deletion cassette. The primers F1/R1 confirmed complete gene replacement, while F2/R2 amplified the “*Up+hph*” fragment for knockout validation (Table S1).

### 2.7. Subcellular Localization of AfFlbD Protein

A 2000 bp upstream sequence of the *AfH2B* gene (promoter) and a 1000 bp downstream sequence (terminator) were retrieved from the *A. flagrans* genome [8]. The *AfH2B* promoter, *AfFlbD*, *GFP*, *AfH2B* terminator, and G418 resistance cassette were directionally assembled into the pUC19 vector with the ClonExpress Ultra One Step Cloning Kit (Vazyme, Nanjing, China). The constructed plasmids were transformed into protoplasts of *A. flagrans*, and AfFlbD localization in chlamydospores was observed after G418 selection.

### 2.8. Hyphal Growth Rate Analysis

WT and knockout strains were cultured on PDA (supplemented with 100 μg/mL hygromycin B for mutants) for 4 days. Mycelial plugs (0.8 cm diameter) were inoculated onto PDA and TYGA media (10 g/L tryptone, 5 g/L yeast extract, 10 g/L glucose, 5 g/L molasses, 15 g/L agar), incubated at 28 °C, and photographed daily for 4 days [9,23]. Colony diameters were measured daily. Three replicates per strain were analyzed using GraphPad Prism 10 (San Diego, CA, USA).

### 2.9. Hyphal Morphology Analysis

WT and mutant strains were grown on PDA with embedded cover slips for 2–3 days. Hyphae were stained with 10 μg/mL Calcofluor White (CFW) (Sigma-Aldrich, St. Louis, MO, USA) for 30 min. Hyphal length was examined using fluorescence microscopy [20,23].

### 2.10. Chemical Stress Tolerance Assay

The WT and knockout strains were cultured on PDA (supplemented with 100 μg/mL hygromycin B for mutants) for 4 days. Mycelial plugs (0.8 cm diameter) were inoculated onto PDA plates containing graded concentrations of the following chemical stressors: sorbitol (0.25, 0.5, 1 M), NaCl (0.1, 0.2, 0.3 M), sodium dodecyl sulfate (0.01%, 0.02%, 0.03%), and H_2_O_2_ (1.75, 2.5, 5 mM). Plates were incubated at 28 °C for 5 days. Colony diameters were measured daily to calculate Relative Growth Inhibition (RGI) [9]. Three replicates per condition were analyzed.

### 2.11. Trap Production and Pathogenicity Analysis

WT and knockout strains were cultured on PDA (9 cm diameter) at 28 °C for 5–6 days. Mycelial plugs were transferred to 250 mL PDB and shaken (180 rpm, 28 °C, 12 h). Hyphae were filtered through sterile funnels, rinsed with sterile water, and resuspended in 7 mL sterile water. Suspensions were spread evenly on 3.5 cm WA plates and incubated at 28 °C for 2–3 days. Synchronized L3 stage *C. elegans* were washed with sterile water. Approximately 300 nematodes were added to fungal lawns. Plates were incubated at 28 °C to induce trap formation [9]. Trap counts and nematode mortality were recorded at 0, 3, 6, 12, and 24 h. Three biological replicates were performed per strain.

### 2.12. Chlamydospore Formation Assay

WT and mutant strains were synchronously cultured for 4 days on PDA (mutants on PDA supplemented with 100 μg/mL hygromycin B). Mycelial plugs (0.8 cm diameter) were inoculated onto 6 cm WA plates and incubated at 28 °C. Chlamydospore production was monitored daily until day 14, when final counts were quantified [9]. Three biological replicates were performed per strain.

### 2.13. Quantitative Real-Time PCR (qRT-PCR) Assay

We performed qRT-PCR assay using the ChamQ Universal SYBR qPCR Master Mix (Vazyme, Nanjing, China) on a Roche LightCycler 480 system (Roche Applied Science, Rotkreuz, Switzerland). The amplification protocol was carried out as previously described [9]. The glyceraldehyde-3-phosphate dehydrogenase (*AfGpd*) gene used as the reference, and relative transcript levels were calculated using the 2^−ΔΔCT^ method [24]. All measurements were performed with at least three biological replicates. Gene-specific primers are listed in Table S1.

### 2.14. Data Analysis

Statistical analyses were performed using GraphPad Prism 10 (San Diego, CA, USA). Significance between groups was determined with Student’s *t*-test, with statistical thresholds defined as follows: * *p* < 0.05, ** *p* < 0.01, *** *p* < 0.001 [9]. The experimental data in figures are presented as mean ± standard deviation (Mean ± SD).

## 3. Results

### 3.1. Gene Structure and Protein Sequence Analysis of AfFlbD

**Figure 1:**
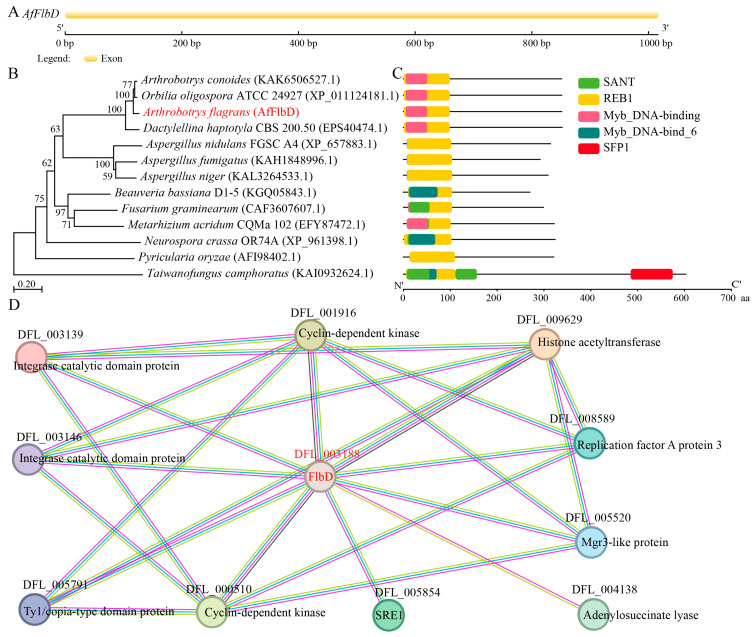
Bioinformatic analysis of *AfFlbD* gene structure, protein sequence, and interacting proteins in *A. flagrans*. (**A**) Gene structure analysis of *AfFlbD*. (**B**) AfFlbD protein phylogenetic analysis. (**C**) Conserved domains of FlbD protein. (**D**). Predicted protein interactions with AfFlbD using the STRING database.

The *AfFlbD* gene comprises a 1017 bp coding sequence lacking introns (Figure 1A), encoding a 338 amino acid protein with a molecular weight of 38.11 kDa and an isoelectric point (pI) of 10.28 (Table S2). Subcellular localization predictions indicated the nuclear localization of AfFlbD (Table S2). A phylogenetic tree constructed using the neighbor-joining method in MEGA X revealed that *A. flagrans* FlbD shares close evolutionary affinity with homologs from *A. oligospora* ATCC 24927, *Dactylellina haptotyla* CBS 200.50, *A. nidulans* FGSC A4, and *A. fumigatus* (Figure 1B). Conserved domain analysis via NCBI CD-Search demonstrated that all FlbD homologs contain the REB1 domain—a DNA-binding motif universally conserved in fungi. The *A. flagrans* FlbD additionally harbors a Myb domain, which aligns with the SANT domains present in most other homologs (Figure 1C). Moreover, protein–protein interaction predictions using the STRING database identified potential binding partners for FlbD, including histone acetyltransferase, cyclin-dependent kinase, integrase catalytic domain protein, Ty1/copia-type retrotransposon domain protein, GATA transcription factor SRE1, adenylosuccinate lyase, Mgr3-like protein, and replication factor A protein 3 (Figure 1D).

### 3.2. AfFlbD Regulates Growth and Stress Responses in A. flagrans

**Figure 2:**
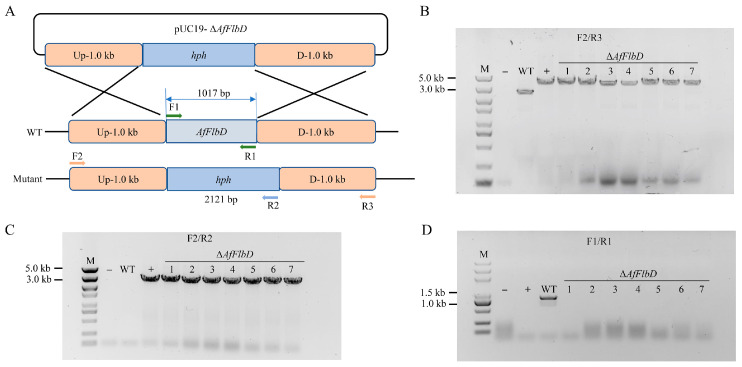
Knockout and validation of *AfFlbD* in *A*. *flagrans*. (**A**) Schematic diagram of *AfFlbD* gene knockout in *A. flagrans*. (**B**–**D**) PCR verification of *AfFlbD* knockout mutants in *A*. *flagrans*.

**Figure 3:**
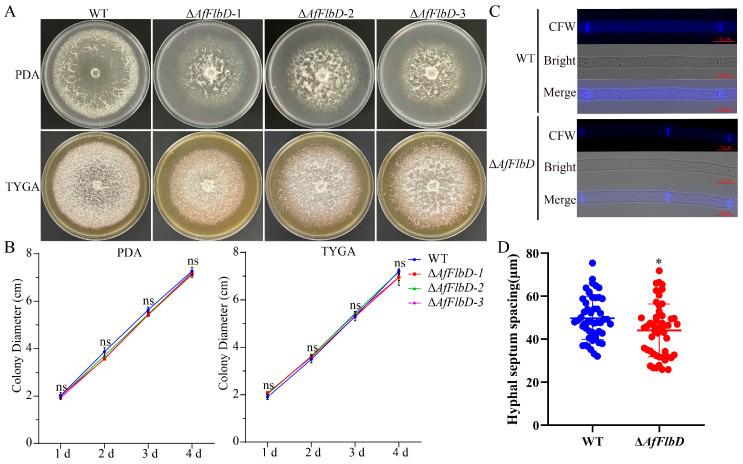
Comparisons of mycelial growth and cell length of the WT and Δ*AfFlbD* strains. (**A**) Colony morphologies of fungal strains cultured at 28 °C for 4 days. (**B**) Comparisons of mycelial growth rates. (**C**) CFW staining of hyphae of WT and Δ*AfFlbD* strains. (**D**) Hyphal septum spacing statistics. (Student’s *t*-test; * *p* < 0.05).

To investigate the biological function of AfFlbD, we generated *AfFlbD* knockout mutants via homologous recombination (Figure 2A), validated by three primer pairs (Figure 2B–D). Complete gene deletion was confirmed in seven mutant strains (Figure 2). To examine the impact of AfFlbD on hyphal growth, the WT and mutants were cultured on PDA and TYGA media for 4 days with daily diameter measurements. All three Δ*AfFlbD* strains showed no significant growth rate differences versus the WT (Figure 3A,B). However, the colonies of the mutants exhibited dense cottony aerial hyphae centrally and sparse peripheral hyphae on PDA (Figure 3A). Calcofluor White (CFW) staining revealed reduced septal spacing in the hyphae of the Δ*AfFlbD* strains (vs. WT), indicating that AfFlbD regulates hyphal cell length (Figure 3C,D).

**Figure 4:**
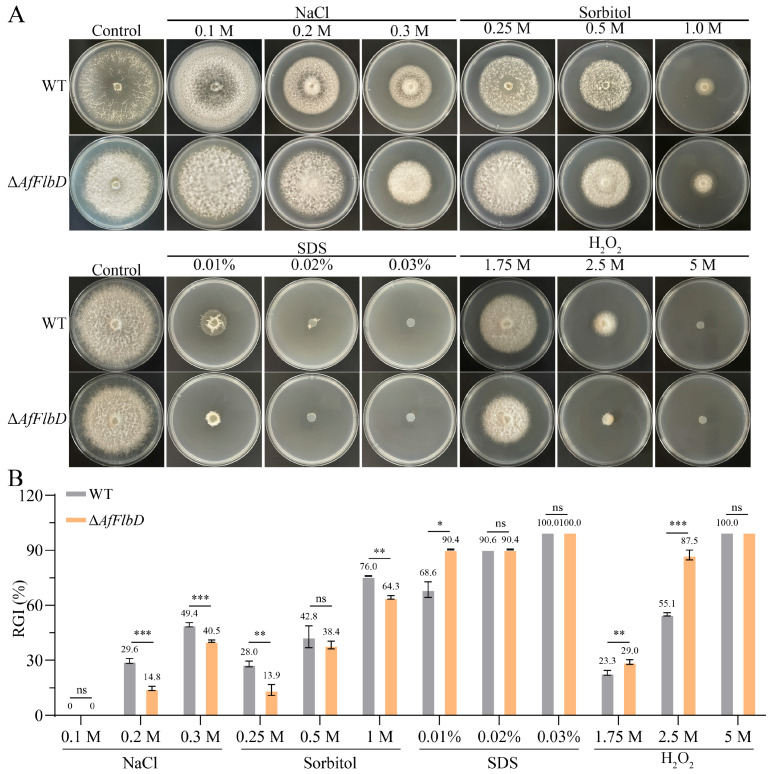
Comparisons of stress responses for NaCl, SDS, sorbitol, and H_2_O_2_ treatment. (**A**) Growth of WT and mutant strains under different concentrations of NaCl, SDS, sorbitol, and H_2_O_2_. (**B**) RGI values of WT and mutant strains under NaCl, SDS, sorbitol, and H_2_O_2_ treatment. (Student’s *t*-test; * *p* < 0.05; ** *p* < 0.01; *** *p* < 0.001).

Furthermore, to investigate the role of AfFlbD in stress adaptation, *A. flagrans* was cultured on PDA supplemented with gradient concentrations of NaCl, sorbitol, sodium dodecyl sulfate (SDS), and H_2_O_2_. All tested stressors inhibited growth in both the WT and Δ*AfFlbD* strains (Figure 4A). Compared with the WT, the RGI values of the Δ*AfFlbD* strains on the media containing NaCl (0.2 M, 0.3 M) and sorbitol (0.25 M, 0.5 M, 1 M) were reduced, indicating that the sensitivity of the Δ*AfFlbD* strains to NaCl and sorbitol was significantly decreased (Figure 4B). In contrast, both the WT and Δ*AfFlbD* strains ceased growth on the medium containing SDS at concentrations of 0.02% and 0.03% (Figure 4A). Compared with WT, the Δ*AfFlbD* strains had higher RGI values on the media containing SDS (0.01%) and H_2_O_2_ (1.75 mM and 2.5 mM), indicating that the sensitivity of the Δ*AfFlbD* strains to SDS and H_2_O_2_ was increased (Figure 4B).

### 3.3. AfFlbD Negatively Regulates the Pathogenicity of A. flagrans

**Figure 5:**
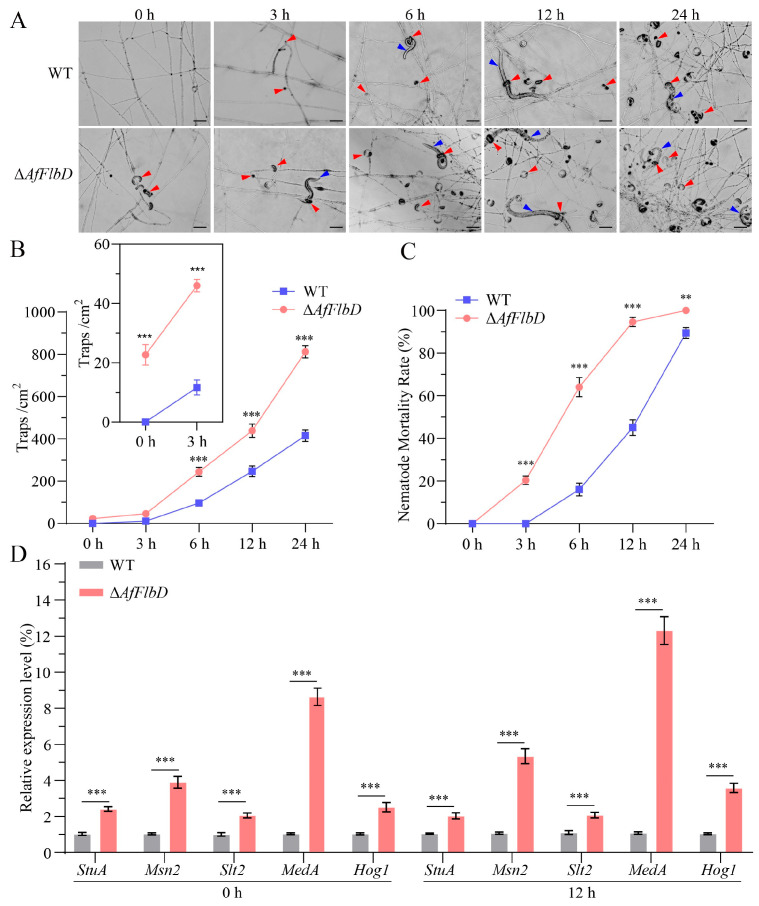
Comparisons of trap formation and nematode predation efficiency of the WT and Δ*AfFlbD* strains. (**A**) Trap formation at different time points (0 h, 3 h, 6 h, 12 h, and 24 h); scale bar: 50 μm. Red triangles: traps; blue triangles: nematodes. (**B**) Numbers of traps at different time points. (**C**) Nematode mortality. (**D**) Analysis of the relative expression levels of five trap-positive regulatory genes in WT and Δ*AfFlbD* strains using qRT-PCR. (Student’s *t*-test; ** *p* < 0.01; *** *p* < 0.001.).

The mycelia of the WT and Δ*AfFlbD* strains were inoculated onto 3.5 cm WA plates and cultured in an incubator at 28 °C for 2–3 days until the mycelia covered the plates. Approximately 300 synchronized *C. elegans* at the L3 stage were added to each plate. The formation of traps and the mortality rate of nematodes were observed and recorded under an optical microscope at 0, 3, 6, 12, and 24 h after inoculation. The results showed that, without nematode induction, the Δ*AfFlbD* strains produced about 22.7 traps (/cm^2^) on WA medium after 3 days of culture, while the WT strain did not produce any traps (Figure 5A,B). After the addition of nematodes, it was observed that the nematodes were captured and killed by the traps produced by the Δ*AfFlbD* strains (Figure 5A). Both the WT and Δ*AfFlbD* strains could be induced to produce traps, but the Δ*AfFlbD* strains produced them significantly faster than the WT strain. The number of traps produced by the Δ*AfFlbD* strains reached about 46.0 (/cm^2^) in 3 h, with a mortality rate of about 20.3%, while the WT strain was almost non-lethal (Figure 5C). As time increased, the number of traps produced by the Δ*AfFlbD* strains was significantly higher than that of the WT strain, about twice as many as the WT strain at 24 h (Figure 5B). The nematode mortality rate of the Δ*AfFlbD* strains reached 94.6% in 12 h after the addition of nematodes, while the WT strain took over 24 h to achieve 89.4% (Figure 5C).

The genes related to trap formation in the nematode-trapping fungus *A. oligospora* have been extensively studied. To explore the mechanism by which AfFlbD negatively regulates the formation of traps, we selected several trap-related genes (*StuA*, *Msn2*, *Slt2, MedA*, and *Hog1*) and compared their expression levels at 0 h and 12 h after the production of traps. In *A. oligospora*, the deletion of *StuA*, *Slt2*, or *MedA* abolished trap formation in mutant strains [25,26,27], whereas the loss of *Msn2* or *Hog1* led to significantly reduced trap production [28,29]. Our results showed that, without nematode induction (0 h), the expression levels of *StuA*, *Msn2*, *Slt2*, *MedA*, and *Hog1* in the Δ*AfFlbD* strains were 2.4-fold, 3.9-fold, 2.0-fold, 9.4-fold, and 2.5-fold higher than those in the WT strain, respectively (Figure 5D), while after 12 h of nematode induction, the same expression levels of in the Δ*AfFlbD* strains were 2.0, 5.3, 2.1, 12.3, and 3.6 times higher than those in the WT strain, respectively (Figure 5D). This confirms that AfFlbD negatively regulates the expression of these genes.

### 3.4. AfFlbD Regulates Chlamydospore Formation in A. flagrans

**Figure 6:**
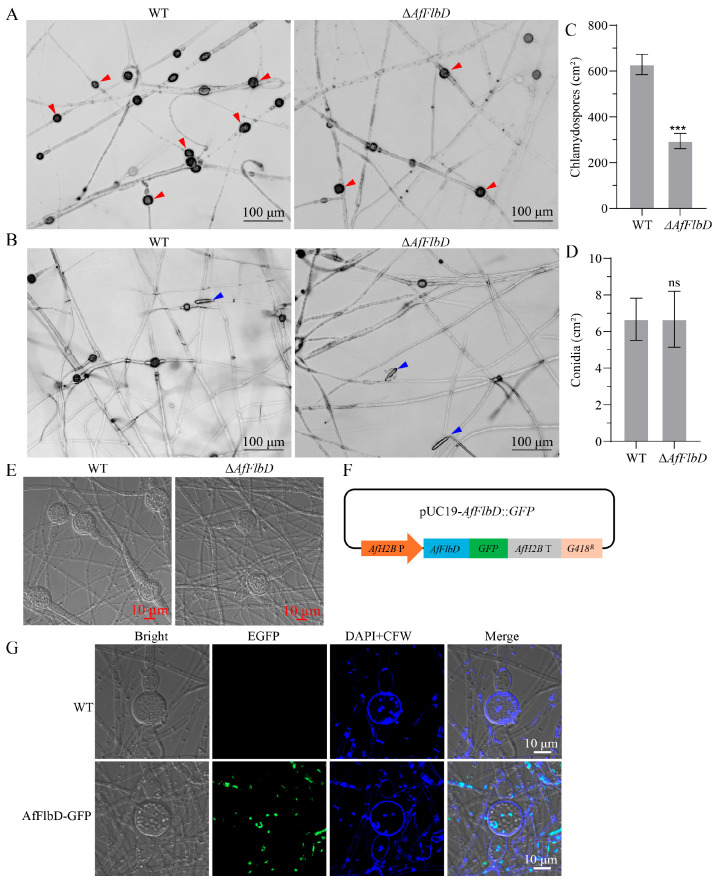
Functional analysis of AfFlbD in chlamydospore and conidia formation. (**A**) The chlamydospores of the WT and Δ*AfFlbD* strains after 14 days of culturing on WA medium. Red triangles: chlamydospores. (**B**) The conidia of WT and Δ*AfFlbD* strains after 14 days of culturing on WA medium. Blue triangles: conidia. (**C**) The number of chlamydospores. (**D**) The number of conidia. (**E**) Comparisons of chlamydospore morphology. (**F**) Schematic diagram of plasmid construction for AfFlbD-GFP fusion protein expression. (**G**) Confocal microscopy analysis of AfFlbD subcellular localization. (Student’s *t*-test; *** *p* < 0.001).

To investigate the regulatory role of AfFlbD in the asexual sporulation of *A. flagrans*, we synchronously inoculated both the WT and Δ*AfFlbD* strains onto 6 cm WA medium for 14 days and observed chlamydospore production under microscopy. The results showed that the *AfFlbD* knockout strains produced both chlamydospores and conidia (Figure 6A,B), but its chlamydospore yield was half that of the WT strain (Figure 6C), with no significant differences in conidial quantity or morphology compared to WT (Figure 6D). Meanwhile, confocal microscopy revealed that the deletion of *AfFlbD* did not alter chlamydospore morphology in *A. flagrans* (Figure 6E). As a transcription factor, AfFlbD was predicted to regulate gene expression in the nucleus (Table S2). Thus, an AfFlbD-GFP fusion protein was expressed in *A. flagrans* using the *AfH2B* gene promoter (Figure 6F). The results demonstrate that the AfFlbD-GFP fusion protein localizes to the nuclei of chlamydospores, thereby regulating chlamydospore development (Figure 6G).

## 4. Discussion

In this study, we conducted a multi-phenotypic comparative analysis of the Δ*AfFlbD* strains and the WT strain, revealing that AfFlbD participate in hyphal development and stress response in *A. flagrans*, in addition to playing crucial roles in chlamydospore formation, trap development, and pathogenicity. Intriguingly, AfFlbD is a positive regulator of chlamydospore formation and a negative regulator of trap production.

Several studies have proved that FlbD is involved in regulation of aerial hyphae morphology but not in hyphal growth rate in fungi [30]. The deletion of the *AfFlbD* gene in *A. flagrans* did not significantly affect hyphal growth rate (Figure 3). However, the Δ*AfFlbD* strains produced abundant cottony aerial mycelia at the colony center, while the periphery exhibited sparser aerial hyphae. Similarly, the deletion of the *FlbD* gene led to very “fluffy-like” hyphae on the colony surfaces of *A. nidulans*, *A. niger*, and *F. graminearum* [3,7,30,31]. This indicates that the functional role of AfFlbD in regulating aerial hyphae morphology is evolutionarily conserved among some fungal species. Additionally, the Δ*AfFlbD* strains exhibited a reduction in hyphal compartment length, indicating that AfFlbD regulates cellular dimensions in *A. flagrans*. Regarding stress response to chemical agents, the Δ*AfFlbD* strains showed reduced sensitivity to NaCl (0.2 M and 0.3 M) and sorbitol but enhanced sensitivity to 0.01% SDS and 2.5 mM H_2_O_2_ (Figure 4). Deletion of the *FlbD* gene in *Beauveria bassiana* also resulted in increased sensitivity to H_2_O_2_ in the mutant strains [32].

The WT strain of *A. flagrans* required nematode induction to form traps on WA medium, whereas the Δ*AfFlbD* strains spontaneously developed traps. After 12 h of interaction with nematodes, the Δ*AfFlbD* strains produced approximately twice as many traps as the WT strain, achieving a 94.6% nematode mortality rate. Within 24 h, the Δ*AfFlbD* strains reached 100% mortality, while the WT strain achieved 89.4%, demonstrating significantly enhanced pathogenicity of the Δ*AfFlbD* strains to nematodes (Figure 5). In contrast, the deletion of the *FlbD* gene in *F. graminearum* leads to a significant reduction in pathogenicity [7]. In *B. bassiana*, knockout of the *FlbD* gene does not affect the pathogenicity of the mutant strains against locusts, whereas the deletion of the *FlbA* or *FlbC* genes significantly reduces pathogenicity [32]. To elucidate the mechanism by which AfFlbD negatively regulates trap formation, this study examined the expression levels of several genes known to positively regulate trap development (*StuA*, *Msn2*, *Slt2*, *MedA*, and *Hog1*) using qRT-PCR. The results revealed that all five genes exhibited higher expression levels in the Δ*AfFlbD* strains compared to the WT strain (Figure 5D), indicating that AfFlbD negatively regulates their expression either directly or indirectly. The formation of traps in nematode-trapping fungi is regulated by multiple genes and pathways, representing a complex biological process [33]. Currently, there is limited evidence in fungi to reveal what downstream genes are regulated by FlbD. Further experiments are required to elucidate the molecular mechanism through which AfFlbD negatively regulates trap formation.

Previous studies have demonstrated that FlbD regulates conidiation: the deletion of *FlbD* in *F. graminearum* significantly reduced conidial production [7]; *FlbD* knockout in *A. niger* markedly decreased conidial yield and even completely suppressed conidiation under certain conditions [30], and FlbD deficiency in *M. oryzae* abolished conidia production [31], while *FlbD* deletion in *A. nidulans* substantially reduced conidial production [3]. Notably, FlbD deletion in *A. flagrans* did not affect conidiation, highlighting regulatory divergence across fungal species. Although existing studies consistently establish the role of FlbD in conidial regulation, no prior research has investigated its involvement in chlamydospore formation. This study reveals that *AfFlbD* knockout delays chlamydospore development and reduces chlamydospore yield to ∼46.7% of WT levels, indicating its critical role for chlamydospore formation in *A. flagrans* (Figure 6).

The molecular mechanism of FlbD-mediated conidiation is relatively well-characterized. In the model fungus *A. nidulans*, FlbD operates downstream of FluG (a developmental activator) but upstream of the transcription factors FlbB, FlbC, FlbE, and BrlA. FluG indirectly activates FlbD by relieving suppression by SfgA (a negative regulator), thereby promoting asexual sporulation [34]. Additionally, FlbB and FlbD form a complex that co-activates *brlA* expression. *FlbD* deletion prevents FlbB from binding to the promoter of brlA—the core conidiation regulator—inhibiting its expression and subsequent conidiation [5]. Conversely, *FlbD* overexpression activates *brlA* and induces the formation of complex conidiogenic structures (including stalks, vesicles, phialides, and conidia) [6]. Critically, the molecular mechanism whereby FlbD regulates chlamydospore formation remains to be elucidated.

Root-knot nematodes (RKNs) infect crops and cause significant agricultural losses annually, while the use of chemical pesticides leads to nematode resistance and poses environmental and food safety concerns [35,36]. Biological control represents an effective and eco-friendly strategy for RKNs management. *A. flagrans* produces traps and stress-resistant chlamydospores, endowing this fungus with substantial research and practical value [17]. In this study, AfFlbD exhibits dual regulatory roles in trap formation and chlamydospore development, providing insights for developing effective *A. flagrans*-based strategies against pathogenic nematodes. Our study provides valuable references for studying chlamydospore development in other biocontrol fungi and pathogenic fungi. Furthermore, investigation of chlamydospore biogenesis in pathogenic fungi (such as *Candida albicans*, *Botrytis cinerea*, and *M. oryzae*) that produce chlamydospores lays crucial molecular groundwork for developing broad-spectrum, eco-friendly control agents against diverse pathogenic fungi.

## 5. Conclusions

In this study, we employed homologous recombination to knockout the *AfFlbD* gene in *A. flagrans* and systematically characterized its biological functions through multi-phenotypic analysis. Our results demonstrate that AfFlbD plays crucial roles in hyphal growth, stress response, pathogenicity, and chlamydospore formation in *A. flagrans*. Significantly, AfFlbD functions as a negative regulator of trap development while serving as a positive regulator of chlamydospore formation. As the first investigation of the FlbD ortholog in a nematode-trapping fungus, this work provides novel insights into the biological functions of FlbD during nematode predation in these fungi.

## Data Availability

The raw data supporting the conclusions of this article will be made available by the authors on request. The *Arthrobotrys flagrans* genome database used in this study is available at the National Center for Biotechnology Information (NCBI) GenBank under the accession number PRJNA917252.

## Supplementary Materials

The following supporting information can be downloaded at: https://www.mdpi.com/article/10.3390/microorganisms13081847/s1, Table S1: Primers used for genetic manipulation; Table S2: The predicted physiochemical properties of AfFlbD in *A. flagrans*.
