# Supplementary material for: FlbD: A Regulator of Hyphal Growth, Stress Resistance, Pathogenicity, and Chlamydospore Production in the Nematode-Trapping Fungus Arthrobotrys flagrans
Source: Microorganisms. 2025 Aug 7;13(8):1847. doi: 10.3390/microorganisms13081847 (PMC12388469; doi:10.3390/microorganisms13081847)
Supplement: Supplementary file 1 [file microorganisms-13-01847-s001.zip › microorganisms-3752216-supplementary.pdf]

Supplementary materials for

FlbD: a Regulator of Hyphal growth, Stress Resistance, Pathogenicity, and Chlamydospore Production in the Nematode-Trapping Fungus *Arthrobotrys flagrans*

Yu Zhang, Shun-Qiao Peng, Wang-Ting He, Fei-Fei Gao, Qian-Fei Shi, Guo-Hong Li*

State key Laboratory for Conservation and Utilization of Bio-Resources in Yunnan, Key Laboratory for Microbial Resources of the Ministry of Education, School of Life Sciences,School of Life Sciences, Yunnan University, Kunming, Yunnan 650091, China

*Correspondence: ligh@ynu.edu.cn (Guo-Hong Li)

Content

Table S1–S2.

Table S1 Primers used for genetic manipulation.

Primers	Target gene (Sequence ID)	Sequence (5′−3′)	Description
FlbD-up-for (F2)	*AfFlbD*	ttcggatcttccagaATGCGATACGAGGTTTGTGATTT	Amplify the 5' homologous
FlbD-up-rev	(EVM02G022510)	ccttcaatatcatcttctgTATTGTAGTTTTTGTAATTTTTGAAAGACC	fragment of *AfFlbD*
FlbD-hyg-for	*hph*	aCAGAAGATGATATTGAAGGAGCATTT	Amplify the *hph* cassette
FlbD-hyg-rev (R2)		ttgtgaggataAAAGAAGGATTACCTCTAAACAAGTGTACC	
FlbD-down-for	*AfFlbD*	tccttctttTATCCTCACAAACCTATTTCCATAATTG	Amplify the 3' homologous
FlbD-down-rev (R3)	(EVM02G022510)	caactgccgttcgacgatatcTCGGTGCTCTTTACTTATATCTGTTGG	fragment of *AfFlbD*
F1	*AfFlbD*	GAATATCACAACTAGTTGGAAC	Amplify the AfFlbD gene
F2	(EVM02G022510)	AATGATACGGTCATCGGAGT	
AfGpd-for	*AfGpd*	CGAGAAGCCCGCCAAGT	qRT-PCR
AfGpd-rev	(EVM05G002080)	AAAGGTGTCGGTCAAAGCAAT	
RT-*AfStuA*-for	*AfStuA*	GCCTCCCGTGGGTCGTATTA	
RT-*AfStuA*-rev	(EVM01G014700)	TCGTAGCGAGTGAGGTGAGGA	
RT-*AfMsn2*-for	*AfMsn2*	CAGACATCCTCTTCCCACCAAT	
RT-*AfMsn2*-rev	(EVM03G012820)	GTCTTCGGAGTCACCCAAGTC	
RT-*AfSlt2*-for	*AfSlt2*	GAACCTACTTGCCCTGCTGAA	
RT-*AfSlt2*-rev	(EVM04G001370)	TCCAACCCACCTGCCAACTCT	
RT-*AfMedA*-for	*AfMedA*	GATGTTTCGCCACCACAATCA	
RT-*AfMedA*-rev	(EVM05G006970)	GCGTAATCCTCCTCCTTTCCTG	
RT-*AfHog1*-for	*AfHog1*	GGAGATGTTGGAGGGAAAGCC	
RT-*AfHog1*-rev	(EVM05G008210)	CTTCTGAGAAAGTGGGATACGC	

Table S2 The predicted physiochemical properties of AfFlbD in *A. flagrans*

Gene	Length (bp)	Exon Number	Intron Number	Amino Acids (aa)	Molecular Weight (kDa)	PI	Subcellular localization
AfFlbD	1017	1	0	338	38.11	10.28	Nuclear
